# Utilization of natural and waste sources for synthesis of cellulose, chitin, and chitosan for a suitable environment

**DOI:** 10.1039/d5ra02896e

**Published:** 2025-07-23

**Authors:** Bipul Mondal Sagar, Md. Mozahidul Islam, Md. Lawshan Habib, Samina Ahmed, Md. Sahadat Hossain

**Affiliations:** a Department of Applied Chemistry and Chemical Engineering, Gopalganj Science and Technology University Gopalganj Bangladesh lawshanhabibsh20@gmail.com; b Department of Applied Chemistry and Chemical Engineering, Noakhali Science and Technology University Noakhali Bangladesh; c Institute of Glass and Ceramic Research and Testing, Bangladesh Council of Scientific and Industrial Research (BCSIR) Dhaka-1205 Bangladesh shanta_samina@yahoo.com saz8455@gamil.com

## Abstract

The growing need for sustainable materials has sparked interest in natural polymers such as cellulose, chitin, and chitosan. This review explored the synthesis routes and various natural and waste-derived sources of these biopolymers. Chitin and chitosan, obtained primarily from crustaceans, insects, and microorganisms, are economically valuable due to their biodegradability and biocompatibility. Chitosan is produced through demineralization, deproteinization, and deacetylation using either chemical methods or microbial fermentation. Cellulose is extracted from agro-waste (*e.g.*, banana peels, rice husks) and textile residues using chemical or mechanical techniques, with processing occurring on nano to macro scales through pre-hydrolysis, bleaching, and pulping. Emphasizing synthesis conditions, environmental implications, and industrial relevance, this review aims to comprehensively understand these polymers' structural characteristics and processing pathways, offering guidance for future research and sustainable waste valorization.

## Abstract 2

The sustainable synthesis of natural polymers such as cellulose, chitin, and chitosan were explored.

## Graphical abstract

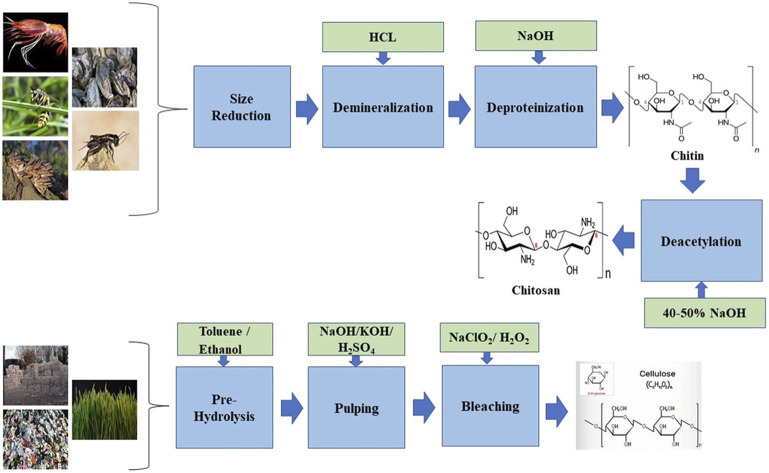

## 1. Introduction

As environmental pollution and climate change escalate, sustainable waste management is crucial. Researchers are developing eco-friendly materials to minimize ecological harm and enhance resource efficiency.^1,2^ Every year, the seafood processing industry generates millions of tons of shell debris, which includes waste from shrimp, lobster, and crabs. The EU handles more than 100 000 MT of shell trash, but India alone produces up to 80 000 MT.^3^ The large volume of waste is difficult for the seafood industry to manage, and they are frequently viewed as an environmental burden. Landfills, incineration, and ocean dumping are examples of disposal techniques that harm ecosystems, cause climate change, and deplete oxygen. Biological decline, ocean acidification, and widespread coral siltation are further environmental problems.^4–6^ Because of its non-toxic, biodegradable, and biocompatible qualities, seafood waste, which is abundant in polysaccharides like chitin and its derivative chitosan, has enormous potential.^6,7^ Chitin is a white, harsh, rigid, and nitrogenous substance regarded as a regenerative raw material.^8^ The second most abundant polymer after cellulose, chitin is present in the exoskeletons of arthropods, yeast, and marine life.^6,9^ Chitins are also mostly found in the exoskeletons of insects, mollusks, fungi, and annelids.^10^ Its yearly biological output is estimated to be between 10^10^ and 10^12^ tons worldwide. Seafood waste is still mostly dumped in landfills or the ocean, causing pollution despite its commercial potential.^3^

Waste textiles, consisting of cellulose, are plentiful but mostly unused. In 2020, 109 million tons of fiber were produced worldwide, with 36% of that amount coming from plant and synthetic cellulosic fibers. The largest portion, 26.2 million tons, was comprised of cotton. Only 14% of post-consumer apparel was recycled in 2017, despite the possibility of recycling; the majority ended up in landfills or incinerators.^11^ In 2010, 75.5 million tons of textile fibers were needed, and by 2030, that number is predicted to rise to 133.5 million tons.^12^ The accumulation of textile waste in landfills favors the spread of diseases and contributes to greenhouse gas emissions.^13^ European households generated over 200 million tons of waste in 2014. Despite recycling systems, much paper waste ends up in mixed municipal solid waste (MSW). The EU set targets for 55% MSW recycling by 2025 and 65% by 2035, aiming to reduce landfilling and enhance sustainability through stricter packaging waste rules.^14^

The primary component of plant cell walls, cellulose, keeps plants rigid and straight. The first source of cellulose was plants. The French chemist Anselme Payen used plants as a source of cellulose. He extracted cellulose from plants and determined its chemical formula in 1838.^15^ Based on size, cellulose can be divided into two categories: microcellulose and nanocellulose.^16^ Nanocellulose (at least one dimension ≤100 nm) is further subdivided into three principal types: bacterial nanocellulose (BNC), nanocrystalline cellulose (NCC or CNC), and nano- or micro-fibrillated cellulose (NFC or MFC).^17,18^ BNC is biosynthesized by certain bacterial species, such as *Komagataeibacter xylinus*, and is known for its exceptional purity, high crystallinity, and three-dimensional nanofiber network, making it ideal for biomedical applications due to its outstanding biocompatibility and mechanical strength.^19^ NCC is obtained through acid hydrolysis of cellulose fibers, producing rod-shaped, highly crystalline particles typically 100–500 nm in length and 5–20 nm in width.^20^ In contrast, NFC consists of long, flexible fibrils containing both crystalline and amorphous domains. It is produced *via* mechanical shearing, often assisted by enzymatic or chemical pretreatments. It is characterized by high viscosity, water-holding capacity, and potential applications in coatings, packaging, and biocomposites.^21^

There are three methods to extract cellulose: mechanical, chemical, and bacterial methods. Among the mechanical cellulose extraction techniques are steam explosion, crushing, grinding, and high-pressure homogenization. Alkali treatment, chemical retting, degumming, and acid retting are examples of chemical extraction techniques.^22^ Cellulose can also be extracted from rice husk,^23^ rice straw,^24^ sugarcane bagasse,^25^ cotton stalk,^26^ sisal fiber,^27^ mengkuang leaves,^28^ kenaf,^29^ jute,^30^ coconut coil,^31^ pineapple crown leaves,^32^ pineapple leaves,^33^ banana peel,^34^ alfa grass,^35^ municipal grass^36^ Native African Napier grass,^37^ sabai grass,^38^ bamboo,^39^ barks of mulberry,^40^ wheat straw,^41^ corncob,^42^ poplar trees wood,^43^ soybean,^44^ lemon peel,^45^ jackfruit peel^46^ Palm oil empty fruit bunches,^47^ carrot peel,^48^ onion peel,^49^ pumpkin peel,^50^ tomato peel,^51^ potato residues,^52^ abaca pulp^53^*etc.* Cellulose can also be extracted from waste paper^54^ and textile waste fabrics.^55^

Chitin, chitosan, and cellulose are natural biopolymers widely studied for their structural and functional properties. These materials share several characteristics, including their polysaccharide nature, biodegradability, and applications in biotechnology.^56^ Despite structural differences, these biopolymers exhibit hydrophilic properties, influencing their solubility and applications. Chitosan, unlike chitin and cellulose, is soluble in acidic solutions, which enhances its usability in biomedicine and environmental science.^57^ Chitosan is widely applied in wastewater treatment, removing heavy metals and contaminants, while cellulose derivatives contribute to sustainable agriculture.^58,59^ Additionally, all three materials are also used in wound healing and drug delivery due to their ability to form films and absorb impurities.^60^ Their biodegradability and film-forming ability enable environmentally friendly packaging and bioplastics production.^61^ In the food industry, chitosan-based coatings improve food preservation, while cellulose enhances dietary fiber content.^62^ In cosmetics, chitosan aids skin hydration and hair care, whereas cellulose-based polymers serve as anti-aging agents.^63,64^

While several reviews have extensively discussed the chemistry, modifications, and applications of cellulose, chitin, and chitosan, limited comprehensive analysis focuses specifically on their extraction from diverse natural and industrial waste sources. This review fills that gap by providing a comparative overview of biological and chemical extraction techniques applied to various waste materials, with detailed tabular data on extraction parameters, sustainability concerns, and source-specific challenges.

## 2. Manuscript collection and search strategy

A comprehensive and methodical literature search was conducted to support the development of a focused and evidence-based review on the extraction of cellulose, chitin, and chitosan from both natural and waste-derived sources. The aim was to identify high-quality, peer-reviewed studies that contribute meaningfully to environmental sustainability and waste valorization. Special attention was given to publications that described chemical and biological extraction techniques and assessed their efficiency, scalability, and ecological impact.

To ensure rigor and transparency, well-defined inclusion and exclusion criteria were applied, including the relevance to the review topic and publication within the last 10 to 15 years. A multi-stage screening process was employed, and the search encompassed several major scientific databases. The complete methodology, including search parameters, keyword strategies, filters applied, and selection criteria, is summarized in Table 1.

**Table 1:** Literature search methodology

Step	Description
Databases used	ScienceDirect, Royal Society of Chemistry, Scopus, PubMed, SpringerLink, ACS, and Google Scholar
Search keywords	Chitin extraction from waste, chitin and chitosan extraction method, chitosan extraction biological method, green extraction of chitosan
	Cellulose from cotton waste, chemical *vs.* biological extraction of chitin/chitosan, cellulose from newspaper, cellulose extraction from agricultural waste, cellulose from plant source, cellulose extraction method
Time Frame	Publications from 2010 to 2024
Language	English only
Inclusion criteria	• Peer-reviewed journal articles
	• Experimental and review papers with detailed methodology
	• Studies focused on the extraction of chitin, chitosan, or cellulose from natural or waste sources
	• Studies describing detailed extraction methodologies (chemical, biological, or combined)
	• Research involving green, eco-friendly, or sustainable extraction techniques
	• Papers that include quantitative data (*e.g.*, yield, purity, deacetylation degree, crystallinity index, *etc.*)
	• Articles discussing novel or advanced technologies (*e.g.*, enzymatic, microbial, or nano-based extraction methods)
	• Review papers that consolidate extraction processes, challenges, and sustainability considerations
Exclusion criteria	• Non-English papers
	• Duplicate entries
	• Articles without relevant extraction data
	• Non-peer–reviewed sources (*e.g.*, blog posts, conference abstracts, patents)
	• Focused only on applications
Screening process	• Titles, abstracts, and conclusions were screened first
	• Full texts were reviewed for relevance and quality
Number of studies reviewed	Approx. 200 articles were reviewed and evaluated

## 3. Chitin and chitosan

Chitin has two forms. The deacetylated form of chitin is known as chitosan. Chitin and chitosan are composed of two polymers collectively known as glycosaminoglycans. Glycosaminoglycans are a group of glucosamine and acetylglucosamine. A polymer composed only of acetylglucosamine is called chitin, and one composed only of glucosamine is called chitosan. Chitin can be converted into chitosan. As chitin is composed of an unbranched *N*-acetyl-d-glucosamine chain, and chitosan consists of only d-glucosamine. The *N*-acetyl part is absent in chitosan. Therefore, if the acetyl groups are removed from chitin, it converts into chitosan. That is how chitosan is produced.^65^ We can define chitin and chitosan based on solubility. Based on the property, whether it is soluble or not in 0.1 M acetic acid, chitin and chitosan should be categorized using the terminology suggested by the European Chitin Society (EUCHIS); chitosan is the name for the soluble substance, while chitin is the term for the insoluble one.^66^

### 3.1 Chemical structure

#### 3.1.1 Chitin

**Fig. 1:**
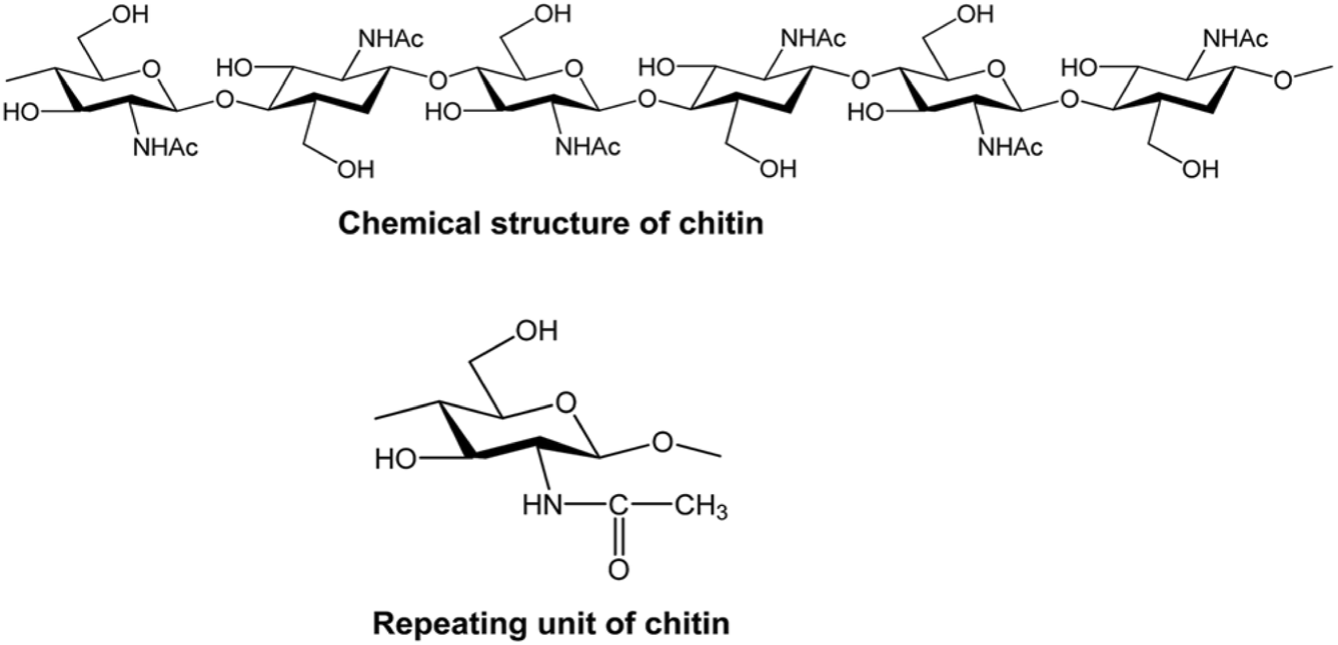
Chemical structure of chitin.^71^

While researching the cuticle of some insects, Antoine Odier discovered it in 1823. He named it chitin, derived from the Greek word “chiton”, which means cloak or wrap. Children discovered nitrogen in 1824 by removing chitin from the elytra of May bugs. In 1843, nitrogen was also observed by Payen, Fischer, and Leuchs. Its primary component, according to Karrer and Zechmeister, is *N*-acetylglucosamine. Early in the 20th century, Meyer and Pankow used X-ray diffraction experiments to validate the structure of chitin.^67^ Chitin is one of the most easily available natural polymers.^67–69^ The structure of chitin and cellulose is quite similar. The only difference is that, unlike cellulose at carbon number 2, chitin has the *N*-acetyl group (in Fig. 1), whereas cellulose has a hydroxyl group there.^70^

Chitin comes in three different forms: α, β, and γ. The α-form is abundantly available and mostly found in crab and shrimp shells. Commercially, α and β chitin/chitosan are both accessible. The alignment of the α-chitin chains is antiparallel, accompanied by strong hydrogen bonds, which increases their stability. The γ-form of chitin has two parallel and one antiparallel strand, while the β-form, which is primarily found in mollusks like squid, is oriented in parallel. When γ-chitin is treated with lithium thiocyanate, the reagent interacts with the chitin chains, breaking weaker hydrogen bonds and facilitating the rearrangement of the molecular structure. This process leads to the transformation of γ-chitin into the more stable α-chitin form.^72^

#### 3.1.2 Chitosan

**Fig. 2:**
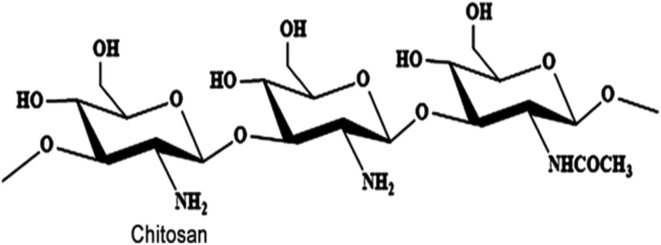
Chemical structure of chitosan.^74^

Chitosan is a polymer made entirely of glucosamine. It is a naturally occurring biopolymer derived from chitin, the primary structural element of squid pens, shrimp and crab shells, and the cell walls of some fungi.^72^ Rouget discovered that heating chitin in an alkaline medium produced a substance soluble in organic acids in 1859. Hoppe-Seyler named this substance chitosan in 1894, but its chemical makeup was not determined until 1950.^70^ High temperature and strong alkali treatment are required for the deacetylation process of chitin to convert it into chitosan (Fig. 2).^73^

The deacetylation process results in converting the acetamide groups of chitins into primary amino groups. This polysaccharide is natural, biocompatible, recyclable, non-toxic, and antibacterial. It comes in various forms, including solution, powder form, flake, fiber, and film.^75^

### 3.2 Elemental composition of chitin and chitosan polymer

**Table:** 

Name	Carbon %	Nitrogen %	Hydrogen %	Ref.
Chitin	47.3	6.5	6.9	76
Chitosan	44.11	7.97	6.84	72

## 4. Extraction process of chitin and chitosan

Chitin and Chitosan are extracted mainly from crab shells and sea shrimp crustaceans. This extraction is done using (1) Chemical method and (2) Biological method.^77^

### 4.1 Chemical method

**Fig. 3:**
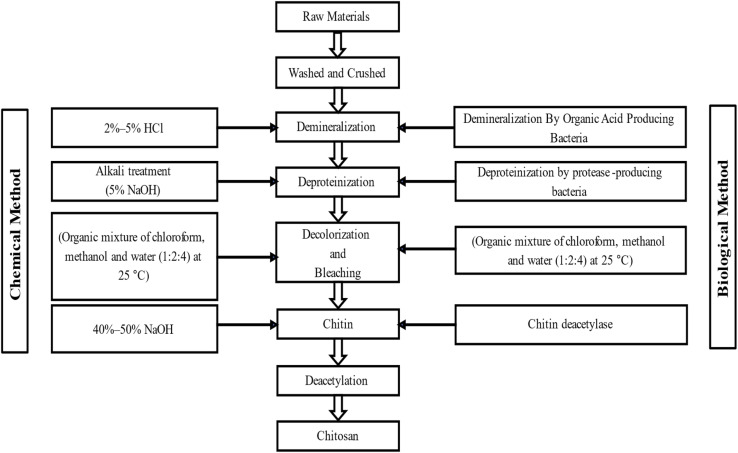
Flow sheet of the extraction process of chitin and chitosan.^8,79^

Deproteinization, demineralization, and discoloration are the three main steps of the chemical extraction method. Among all extraction methods, the chemical extraction method is mostly used. Deproteinization is mainly the depolymerization of biopolymers, which breaks down the bond between the chitin and protein. Demineralization is done using strong acids like H_2_SO_4_, HCl, HCOOH, HNO_3,_ and CH_3_COOH to remove calcium carbonate and other minerals (Fig. 3).^77^ Generally, calcium carbonate reacts with acids and produces salt, water molecules, and carbon dioxide (in eqn (1)).^78^ Then the discoloration process is done to get a colorless product by removing pigments like β-carotene and astaxanthin. Mainly, acetone is used in this process.^8^

1 2HCl + CaCO_3_ → CaCl_2_ + H_2_O + CO_2_↑ (ref. **78**)

Suneeta Kumari *et al.* used three natural waste sources to extract chitin. They used *Labeo rohita* as fishery waste, the shell of *Crangon crangon* as Crab waste for the source of chitin.^79^ At first, they removed the protein matter from their sources (3% NaOH, 30 min, 80 °C), removed minerals, and extracted chitin by treating them with 3% HCl for 30 minutes at room temperature. Then they deacetylated chitin to produce chitosan (40% KOH, 6 hours, 90 °C). The degree of deacetylation of chitosan obtained from fish, shrimp, and crab was 75%, 78%, and 70%, respectively.^79^

Microwave irradiation can be incorporated with the chemical extraction process to improve the quality and reduce the time required for the chemical reactions. El Knidri H. and El Khalfaouy combined microwave heating with the conventional chemical extraction process. The deacetylation process took around 24 minutes with microwave heating, whereas the traditional method needed 6.5 hours. The degree of deacetylation with microwave irradiation was slightly higher than that of the chitosan obtained by the traditional heating method (82.73% and 81.50%, respectively). Moreover, the molecular weight of the microwave-heated chitosan was higher.^80^

### 4.2 Biological methods

Besides chemical methods, biological methods are also available to prepare chitosan. There are two types of biological methods: (1) Enzymatic method (2) Fermentation method.^70^

This method is more environmentally friendly as it requires no harsh chemicals. Acid-producing bacteria and protease-producing bacteria are mainly used in this process for demineralization and deacetylation. This process is time-consuming compared to other processes. The efficiency and quality are low as well. That is why this is a costly process with less industrial usage.^81^

#### 4.2.1 Enzymatic method

There is no difference between chemical and enzymatic methods for the demineralization process. The biological extraction process involves enzymes and microorganisms to extract chitin from its sources. The enzyme protease does the deproteinization process (Fig. 3). Pepsin, trypsin, papain, alkaline, pancreatin, *etc.*, are some commonly used proteases. The primary sources of these proteases are plants, microorganisms, and animals. Based on some parameters, biological extraction is better than the chemical extraction process because it is more environmentally friendly and does not change the structure of chitin.^8^

In this method, acid removes the CaCO_3_ in the shell.^70,82^ As this method uses proteinases for enzymatic deproteinization and deacetylases extracted from microbes and fish intestines for deacetylation, the application of elevated temperatures and strong alkali treatment is eliminated. This makes the method more environmentally friendly.^83,84^ For example, Alcalase is obtained from *Bacillus licheniformis*. This method follows complex reaction mechanisms. Using an acid/base reaction mechanism, CE4 (a member of the carbohydrate esterase enzyme family) may remove *N*-acetyl functional groups when metal ions (often zinc or cobalt) are attached to the enzyme's reaction site.^85^

Furthermore, this method's degree of deacetylation value is much lower, and this method is less effective than chemical methods because the enzymatic method cannot remove the last 10% of the proteins during deproteinization.^86^ Fermentation techniques have been created as an option for solving the problem of the high cost of enzymes. This is because microorganisms may multiply while secreting enzymes into reactors under optimal reaction conditions, hence lowering the cost of enzymes.^86,87^

#### 4.2.2 Fermentation method

The fermentation process is used in place of the enzymatic method for making chitin or chitosan. Various types of bacteria are used for this fermentation process. Bacteria that produce lactic acid are used in the lactic acid fermentation process. Also, bacteria that produce other organic acids rather than lactic acid are used in non-lactic acid fermentation. These lactic or other types of organic acids produced by the bacteria cause the demineralization of raw materials (Fig. 3). When calcium carbonate and lactic acid combine, calcium lactate is produced, which may be precipitated and eliminated.^70^ Moreover, the deacetylation process to convert chitin into chitosan can be carried out by acetyl xylan esterase.^66^

Aranday-García used *Lactobacillus brevis* (20% v/w) and *Rhizopus oligosporus* for the demineralization and deproteinization, respectively.^88^ They inoculated shrimp shells with *Lactobacillus brevis* and sucrose (10% w/w) for 2 hours at 30 °C. Following that, they added *Rhizopus oligosporus* and glucose (10% w/w) and incubated for 72 hours. The culture was then kept for fermentation for 8 days. After 8 days of fermentation, the chitin was rinsed with water and dried. The remaining mineral and protein content was removed by the conventional method (HCl 0.4 N 25 °C for 2 hours and NaOH 0.5 N 25 °C for 2 hours). The final product was obtained by rinsing the sample with distilled water and drying it for 2 days at 40 °C. The efficiency of deproteinization and deacetylation was 96.8% and 97.5%, respectively.^8^

### 4.3 Combined chemical and biological methods

A combination of chemical and biological processes can extract chitin and chitosan. Younes and Hajji used enzymatic protease for deproteinization and conventional acid alkali methods for demineralization and deacetylation because chitin produced by deproteinization by enzymatic proteases has higher molecular weights compared to chitin which is obtained by chemical deproteinization.^82^ Several microbial and fish alkaline proteases were used for the deproteinization of shrimp shells. They digested shrimp shells with enzymes for 3 hours and stopped the reaction by heating the solution for 20 minutes at 90 °C to inactivate the enzymatic action. The solid product obtained from the enzymatic action of *Bacillus mojavensis* A21 and *Balistes capriscus* proteases was treated with HCl (1.5 M, 25 °C, 6 hours) for demineralization. After that, they were treated with NaOH (12.5 M, 12 hours, 50 °C) for deacetylation. The efficiency of deproteinization by *Bacillus mojavensis* A21 and *Balistes capriscus* was 77 ± 3% and 78 ± 2%, respectively.^82^

### 4.4 Sources of chitin and chitosan

The extraction processes described in Sections 4.1 to 4.3 serve as the basis for evaluating how chitin and chitosan can be obtained from different biological sources. This section explores how these methods are applied to various organisms, emphasizing waste.

There are several sources from which chitin may be extracted. Chitin is commercially extracted from the shells of shrimp, crabs, crab fish, and krill. According to some recent research, other sources of chitin include insects, mushrooms, coral, and resting eggs from crustaceans.^79^

#### 4.4.1 Shrimp sources

The contents of shrimp biowaste and fish scales are 15–30% chitin, 30–65% protein, 30–50% minerals, and 10–20% calcium, respectively.^79,82^ Strong acids and bases can be used to eradicate proteins and calcium carbonate.^82^ The heads, shells, and tails of shrimp comprise 40–50% of their weight worldwide. Environmental problems arise because only 5% of it is used for animal feed, and the remainder is thrown away.^89,90^

In Table 2, the main chemical is NaOH in the deproteinization step to extract chitin and chitosan from shrimp sources, whereas HCl is used in the demineralization step. The acetyl group is removed using the strong alkali reagent NaOH in the deacetylation step. During deproteinization, the best conditions for obtaining the he maximum chitin yield (36.7–42.1%) and chitosan (64.1–81.9%) were determined to be 10% w/v NaOH at 100 °C for one hour. Raising the NaOH concentration to 48% at room temperature produced a much lower chitosan yield (4.09%), suggesting that milder conditions are better for effective protein removal. To get the maximum yield percentage, 6% (w/v) HCl is used for 2 hours in the demineralization step, and 50% (w/v) NaOH at 60 °C for 4 hours is applied in the deacetylation step. However, the yield percentage of chitosan is the lowest, at 4.09%, when chitin is converted to chitosan using 48% w/v NaOH at room temperature for 48 hours. Chitin and chitosan yield percentage differences are less significant when 50% (w/v) NaOH is employed at 120 °C; they are 30% and 35%, respectively.

**Table 2:** Extraction methods, conditions, and sources of chitin and chitosan from shrimp shell waste

Deproteinization				Demineralization				Deacetylation				Yield		References
Reagent	Strength	Duration	Temp. (°C)	Reagent	Strength	Duration	Temp. (°C)	Reagent	Strength	Duration	Temp. (°C)	Chitin %	Chitosan %	
NaOH	15% (w/v)	3 h	65	HCl	1 N	2 h	—	NaOH	65% (w/v)	1 h	100	—	—	91
NaOH	10% (w/v)	2 h	80	HCl	3 M	2 h	75	NaOH	50% (w/v)	2.5 h	100	—	—	80
NaOH	3% (w/v)	30 min	80	HCl	3% (w/v)	30 min	25	KOH	40% (w/v)	6 h	90	—	—	79
NaOH	2.5 M	20 min	121	HCl	1.5 M	6 h	25	NaOH	12.5 M	4 h	140	—	—	82
NaOH	2 N	2 h	50	HCl	2 N	2 h	25	NaOH	50% (w/v)	1 h	121	14.72 ± 0.57	12.03 ± 0.46	92
NaOH	1 M	24 h	—	HCl	1 M	—	—	NaOH	50% (w/v)	—	—	—	—	93
NaOH	0.5% (w/v)	30 min	100	HCl	1.5 N	1 h	25	NaOH	42% (w/v)	1.5 h	95	—	—	94
NaOH	4% (w/v)	21 h	25	HCl	4% (w/v)	12 h	25	NaOH	50% (w/v)	3 days	40	—	—	94
NaOH	3 M	75 min	25	HCl	1 M	75 min	25	NaOH	50% (w/v)	1.5 h	90	30	35	89
NaOH	2.5 N	6 h	65	HCl	1.7 N	6 h	25	NaOH	50% (w/v)	—	120	—	17.5	95
NaOH	2 and 4% (w/v)	1 h	100	HCl	1% (w/v)	24 h	—	NaOH	50% (w/v)	2 h	100	—	34	96
NaOH	1 M	—	105–110	HCl	1 M	—	25	NaOH	—	>20 h	—	21.53 brown shrimp	—	97
												23.72 pink shrimp		
NaOH	10% (w/v)	1 h	100	HCl	6% (w/v)	2 h	—	NaOH	50% (w/v)	4 h	60	36.7 to 42.1	64.1 to 81.9	98
NaOH	2 N	4 h	70	HCl	3.25 N	4 h	25	NaOH	8.75 N	75 h	25	—	—	74
	1.25 N	24 h	90	HCl	1.3 N	24 h	25		17.5 N	75 h	25			
	1.25 N	3 h	70	HCl	0.32 N	24 h	25°		12.5 N	12 h	100			
	1 M	24 h	25	HCl	1 N	24 h	25		50% (w/v)	24 h	25			
				NaOH	1 N	24 h	25							
NaOH	1 M	—	70	HCl	1 M, 0.25 M	24 h	25	NaOH	45% (w/v)	—	110	19.13	—	99
NaOH	2 M	48 h	25	HCl	1 M	—	25	NaOH	48% (w/v)	48 h	25	—	4.09	99
NaOH	0.5 M	18 h	25	HCl	1 M	12 h	—	NaOH	—	5–6 h	—	—	—	100
NaOH	4% (w/v)	24 h	25	HCl	4% (w/v)	24 h	25	NaOH	65% (w/v)	3 days	25	—	46	8
NaOH	5% (w/v)	2 h	60	HCl	0.5–1% (w/v)	6 h	25	NaOH	25% (w/v)	2 h	80	—	—	101
									50% (w/v)	5 h	100			
										10 h				
NaOH	0.68 M	16 h	30	HCl	0.68 M		30	NaOH	25 M	20 h	75	—	—	102

The most commonly employed reagents in the chemical extraction of chitin and chitosan are NaOH for deproteinization and deacetylation, and HCl for demineralization. However, using these strong acids and bases raises serious environmental concerns, as they are corrosive, hazardous to handle, and produce toxic effluents. These effluents often require extensive neutralization and treatment before disposal.

#### 4.4.2 Insect sources

Since chitin is mainly sourced from marine waste, the market demand for it now surpasses the supply chain.^103^ The over two million insect species found globally comprise 95% of the animal kingdom. Only a small number of species, such as *Bombyx mori*, *Bombus terrestris*, *Musca domestica*, *Holotrichia parallela*, *Hogna radiata*, and *Geolycosa vultuosa*, have been found to have chitin.^104^ The alpha form of insect chitin has physicochemical characteristics similar to crustacea, such as the shells of shrimp and crabs. Chitin from insects is easier to extract and more ecologically friendly since it includes less calcium carbonate (<6%) than that from crustaceans (30–50%). Additionally, it maintains greater degrees of polymerization with enhanced mechanical, gelling, biological, and flexible qualities. Chitin extraction and its conversion into bioproducts or biomaterials from different insect species and body sections have been the subject of several articles during the last five years.^103^

In Table 3, the maximum amount of chitin can be extracted from *Apis mellifera,* and the percentage range is 51 to 77.2%. At this maximum quantity, 1 M HCl and 1 M NaOH are used for demineralization and deproteinization, respectively. However, the chitin percentage of *Hylobius abietis* is 27.9%, below the maximum amount of chitosan derived from its chitin, which is 86.2%. This source uses 2 M NaOH for 2 hours in the deproteination stage and 22 M NaOH for 4 hours at 100 °C in the deacetylation phase.

**Table 3:** Chitin and chitosan from insect biomass: species, pretreatment approaches, and process parameters

Insect species	Deproteinization				Demineralization				Deacetylation				Yield		References
	Reagent	Strength	Duration	Temp. (°C)	Reagent	Strength	Duration	Temp. (°C)	Reagent	Strength	Duration	Temp. (°C)	Chitin %	Chitosan %	
*Gryllus bimaculatus*	NaOH	1 M	3 h	95	—	—	—	—	NaOH	19–25 M	15 h	—	5.1	41.7 (from chitin)	105
*Dociostaurus maroccanus*	NaOH	2 M	18 h	50	HCl	2 M	1 h	55	NaOH	22 M	4 h	150	12–14	81.7 (from chitin)	106
*Acheta domesticus*	NaOH	1 M	—	95	C_2_H_2_O_4_	0.1 M	3 h	25	—	—	—	—	4.3–7.1	2.3–5.8 (from biomass)	107
*Apis mellifera*	NaOH	1 M	6–64 h	80	HCl	1 M	1 h	25	—	—	—	—	51.–77.2	—	108
*Hermetia illucens*	NaOH	2 M	2 h	80	CH_2_O_2_	0.5 M	1 h	25	NaOH	12 M	6-Mar	120–140	31–35	8–16 (from biomass)	109
*Agabus bipustulatus*	NaOH	1 M	18 h	110	HCl	1 M	1 h	90	NaOH	22 M	2 h	120	14–15	71 (from chitin)	110
*Anax imperator*	NaOH												11–12	67 (from chitin)	
*Ranatra linearis*	NaOH												15–16	70 (from chitin)	
*Notonecta glauca*	NaOH												10–11	69 (from chitin)	
*Celes variabilis*	NaOH	4 M	20 h	150	HCl	4 M	2 h	75	—	—	—	—	6.6–9.9	—	111
*Melanogryllus desertus*	NaOH	4 M	20 h	150	HCl	4 M	2 h	75	—	—	—	—	4.7–7.3	—	111
*Decticus verrucivorus*													10–11.8	—	
*Leptinotarsa decemlineata*	NaOH	2 M	16 h	80–90	HCl	2 M	2 h	65–75	NaOH	19 M	3 h	100	Jul-20	72 (from chitin)	104
*Bombyx eri*	NaOH	1 M	24 h	80	HCl	1 M	0.6 h	80	—	—	—	—	3.3	—	103
*Melolontha melolontha*	NaOH	1 M	18 h	150	HCl	4 M	—	75	—	—	—	—	—	—	112
*Vespula germanica*	NaOH	4 M	18 h	150	HCl	2 M	2 h	75	—	—	—	—	—	—	113
*Vespa crabro*													—	—	
*Argynnis pandora*	NaOH	2 M	24 h	50	HCl	2 M	24 h	50	—	—	—	—	22	—	114
*Hermetia illucens*	NaOH	1.9 M	2 h	50	HCl	0.5 M	2 h	25	NaOH	19 M	2 h	100	46	80 (from chitin)	115
*Hylobius abietis*	NaOH	2 M	2 h	—	HCl	2 M	—	25	NaOH	22 M	4 h	100	27.9	86.2 (from chitin)	116
*Drosophila melanogaster*	NaOH	3 M	20 h	70	HCl	2 M	3 h	40	NaOH	22 M	48 h	150	7.8	71 (from chitin)	117
*Calliptamus barbarus*	NaOH	1 M	21 h	80–90	HCl	1 M	30 min	100	NaOH	19 M	2 h	130	20.5	74 (from chitin)	118
*Oedaleus decorus*													16.5	75 (from chitin)	
*Melontha* sp.	NaOH	2 M	20 h	100	HCl	2 M	20 h	60	—	—	—	—	—	—	119
*Musca domestica*	NaOH	1.25 M	3 h	95	HCl	2 M	3 h	25	NaOH	19 M	05-Mar	95–105	7.7–8.5	6.8	120
*Cicada*	NaOH	1 M	—	80	HCl	1 M	2 h	30	NaOH	22 M	8 h	100	—	28.2	81
*Bombyx mori*														3.1	
*Grasshopper*														5.7	
*Tenebrio molitor*														2.5	
*Gryllus bimaculatus*	NaOH	1.25 M	3 h	95	HCl	2 M	3 h	25	NaOH	19 M	3 h	100	2.4	1.8	81
*Catharsius molossus*	NaOH	4 M	6 h + 12 h	90 + 25	HCl	1.3 M	30 min + 12 h	80 + 25	NaOH	18 M	24 h + 7 h	25–95	24	—	121
*Calosoma rugosa*	NaOH	1 M	8 h	100	HCl	1 M	25	—	NaOH	19 M	8 h	100	5	—	122
*Apis mellifera*													2.5	—	
*Brachystola magna*	NaOH	1 M	24 h	82	HCl	1 M	30 min	97	NaOH	15 M	—	105–110	10.4	8.1	123
									NaBH_4_	0.25 g L^−1^		—			
*Bombyx mori*	NaOH	1 M	3 h	80	HCl	1 M	—	100	—	—	—	—	15–20	—	124
*Beetle*									—	—	—	—	15–20	—	
*Musca domestica*	NaOH	1 M	3 h	100	—	—	—	—	NaOH	15 M	4 h	110	—	60–70 (from chitin)	125
*Apis mellifera*	NaOH	15 M	—	—	—	—	—	—	NaOH	19 M	1 h	150	—	—	126
*Cryptotympana atrata*	NaOH	3.7 M	24 h	60	HCl	2 M	24 h	25	NaOH	21 M	4 h	110	—	—	127
*Calliphora erythrocephala*	NaOH	1 M	2 h	50	—	—	—	—	NaOH	19 M	1–4 h	100–120	12.2	66.7 (from chitin)	128
*Clanis bilineata*	NaOH	3.7 M	24 h	60	HCl	2 M	24 h	25	NaOH	21 M	4 h	110	—	—	127
*Bombyx mori*	NaOH	1 M	—	80	HCl	1 M	—	100	NaOH	15 M	—	100	2.6–4.3	—	129
									NaBH_4_	1 g L^−1^					
*Hermetia illucens*	NaOH	1 M	24 h	80	HCl	1 M	—	—	—	—	—	—	—	—	130
*Allomyrina dichotoma*	NaOH	3.7 M	24 h	80	HCl	2 M	24 h	25	NaOH	21 M	9 h	90		83.4 (from chitin)	131
*Mayfly*	NaOH	2 M	—	100	HCl	2 M	—	50	NaOH	22 M	6 h	150	10.2	78.4 (from chitin)	132
*Chrysomya megacephala*	NaOH	1 M	6	95	C_2_H_2_O_4_	0.1 M	3	—	NaOH	25 M	9 h	90	—	26.2	133
*Zophobas morio*	NaOH	0.5 –2 M	20	80	HCl	1 M	30 min	35	NaOH	19 M	30 h	90	—	65–75 (from chitin)	134

#### 4.4.3 Mushroom sources

Fungi and mushroom wastes are some other sources of chitin and chitosan (Table 4). Unlike chitosan derived from shrimp and crab shells, chitosan extracted from mushroom waste requires less harsh solvents and the process is also simpler. No demineralization process is required to extract chitosan from mushrooms, as mushrooms do not contain any significant amount of metal salts. *Agaricus bisporus* is the most consumed mushroom in the USA, and it is also a good source of chitinous biopolymer. Wu, T. and Zivanovic S. extracted chitosan from stalks of White button mushrooms, *A. bisporus*. To remove proteins, alkali-soluble polysaccharides, and other small molecules, the stalks were stirred in NaOH (1 M, 30 minutes, 95 °C). The remaining insoluble part was then separated by centrifugation and washing and treated with 2% acetic acid (95 °C, 6 hours) to extract acid-insoluble chitin. Chitin was then converted into chitosan by the treating acetic acid at a pH of 10. Their yield was up to 27% and the degree of deacetylation was from 75.8 to 87.6%.^115,135^

**Table 4:** Chitin and chitosan recovery from mushroom-based fungal sources: extraction processes and parameters

Mushroom specimen	Deproteinization				Deacetylation					Yield %		References
	Reagent	Strength	Duration	Temp. (°C)	Reagents	Strength	Duration	Temp. (°C)	pH	Chitin	Chitosan	
White *A. bisporus*, brown *A. bisporus, P. ostreatus*	Ethanol	96% (v/v)	15 min	—	NaOH	50% (v/v)	2 h	104	8.5	—	—	139
	Na_2_S_2_O_5_ + HCl	0.5% (m/v)	1 h	25	HCl	1 M	—	—		—	—	
	NaOH	2%	2 h	56	—	—	—	—		—	—	
	NaOH + H_2_O_2_	0.1 M + 3% (v/v)	30 min	45	—	—	—	—		—	—	
White *A. bisporus*	NaOH	1 M	2 h	80	NH_4_OH	37% (v/v)	—	—	9	7.4	—	140
	Acetic acid	2% (v/v)	6 h	95						—	—	
White *A. bisporus*	NaOH	2 M	2 h	100	NaOH	2 M	—	—	10	—	—	136
	Oxalic acid	1% (w/v)	1 h	100						—	—	
	Acetic acid	2% (v/v)	2	90						—	—	
*Pleurotus ostreatus*	NaOH	1 M	3 h	90	NaOH	10 M	3 h	90	9	—	—	137
					Acetic Acid	2% (v/v)	3 h	90		—	—	
*A. bisporus*, *Pleurotus*, *Ostreatus*, *Ganoderma lucidum*	NaOH	1–4 M	15 min	95, 110, 121	NaOH	—	24 h	4	—	—	19.7	141
	Acetic acid, HCl	2%, 6%, 10%	3 h, 6 h, 12 h	60, 95	—	—	—	—	—	—	41.29	
*A. bisporus*	NaOH	1 M	30 min	95	—	—	—	—	10	—	—	135
	Acetic acid	2% (w/v)	6 h	95						13.98	—	
*Pleurotus ostreatus*	NaOH	1 M	15 min	121	NaOH	2 M	—	—	09-Oct	—	—	142
	Acetic acid	0.35 M	5 h	95						—	—	
*A. bisporus*	NaOH	1 M	2 h	80	—	—	—	—	—	—	—	143
	Acetic acid	2% (v/v)	6 h	95						—	—	
*Pleurotus ostreatus*	NaOH	1 N	3 h	100	NaOH	2 N	—	—	12	—	—	138
	Acetic acid	2%	5 h	100								
*A. bisporus*	NaOH	1 N	12 h	40	NaOH	47%	2 h	60	—	—	—	144
	Acetic acid	5%	3 h	90						8.5 ± 1.4	—	
*Pleurotus ostreatus, Schizophyllumcommune*	NaOH	1 M	24 h	25	NaOH	40%	—	—	—	—	1.22	145
											1.73	
*Pleurotus florida, Pleurotus ostreatus*	NaOH	1 M	2 h	45	NaOH	1 N, 2 N	20 min	121	—	—	—	146
							—	—				
					Acetic Acid	2% (v/v)	5 h	95	—	—	—	
*Ganoderma lucidum*	NaOH	1 M	2 h	40	—	—	—	—	—	41	—	147
*Ganoderma lucidum*	NaOH	4 M	2 h	100	NaOH	45%	2 h	60	—	—	—	

Chitosan might be produced using the chitin fibers found in the cell walls of mushrooms. Different mushrooms have been reported to produce chitosan. However, quality mushrooms may not be required, and waste from the mushroom business might be useful.^136^ Fungal cell walls are complex structures composed of polysaccharides, including chitin/chitosan. The exterior layers of most fungi are more varied and adapted to the physiology of a particular fungus, whereas the inner wall layer is composed of branching β-(1,3) glucan, β-(1,6) glucan, and chitin and is alkali-insoluble.^137^ With few members in the Ascomycotina division, mushrooms belong to the higher fungus division, basidiomycetes. With mycelia networks of hyphae, they are recognizable and fleshy. Being saprophytes, mushrooms may grow in a variety of habitats and substrates. Because of their excellent flavor and high protein content, they have been regarded as an important meal for millennia. A few types of mushrooms have also been used medicinally. Tree trunk tissues, fallen logs, or other nutrient-rich substrates can all support the growth of mushrooms.^138^

In the deproteinization stage, chitin and chitosan are extracted from mushroom specimens using NaOH, acetic acid, oxalic acid, C_2_H_5_OH, HCl, and Na_2_S_2_O_5_. Alkaline solutions (NaOH, NH_4_OH) and acidic solutions (CH_3_COOH, HCl) are used to convert chitin into chitosan during the deacetylation step. Temperature and time vary from chemical to chemical.

#### 4.4.4 Annelida sources

There are various kinds of annelida sources from which chitin and chitosan are extracted, including *Egeria radiata*,^148^*Ensis arciatus*,^149^*Pinna deltoides*,^150^*Mytilus edulis*,^151^ Oyster shell,^152^ Chiton shell,^153^*Acanthopleura vaillantii*,^154^ Mussel shell,^102^*Haliotis tuberculate*,^155^*Modiolus modiolus*,^150^*Bellamya jayanica*,^156^*Donax scrotum*,^157^*Murex trapa*,^158^*Anadara granosa*,^159^*Conus inscriptus*,^160^ Snail shells,^161^*Perna viridis*^162^*etc.* Generally, in demineralization and deproteinization steps, HCl and NaOH are used, respectively, and in the deacetylation step, NaOH is used to convert chitin into chitosan.^157–160,163^

In Table 5, 70.67% is the highest amount of chitin that can be extracted from *Pinna deltoides*. Chitin extraction from oyster shells has a 69.65 percent yield, nearly equal to the maximum percentage. The highest chitosan production (85%) is obtained from *Doryteuthis singhalensis*. In the deproteinization stage, protein is removed for two hours at 90 °C using 4 M HCl, and minerals are removed using NaOH in the demineralization stage.

**Table 5:** Extraction of chitin and chitosan from annelid sources: treatment steps and processing conditions

Source	Demineralization				Deproteinization				Deacetylation				Yield (%)		Reference
	Reagent	Strength	Duration	Duration	Reagent	Strength	Duration	Temp. (°C)	Reagents	Strength	Duration	Temp. (°C)	Chitin	Chitosan	
*Egeria radiata*	HCl	4% w/v	12 h	12 h	NaOH	65% w/m	3 days	25	NaOH	4% w/v	24 h	25	—	48.6	148
*Ensis arcuatus*	HCl	20%	16.5 h	16.5 h	NaOH	50%	10 h	90	NaOH	10%	2 h	70	—	19.36	149
*Ensis arcuatus*	HCl	1 M	2 h	2 h	NaOH	—	—	—	—	1 M	5 h	40	—	—	164
*Pinna deltoides*	HCl	1 M	15 min	15 min	NaOH	45%	24 h	110	NaOH	1 M	20 min	100	70.67	45.01	150
*Mytilus edulis*	HCl	4% v/v	12 h	12 h	NaOH	65% w/v	3 days	25	NaOH	4% w/v	24 h	25	—	51.8	151
*Laevicardium attenuatum*													—	43.8	
Oyster shell	HCl	1 N	2 h	2 h	NaOH	50%	1 h	120	NaOH	3 N	2 days	90	30.01	61.1	152
Chiton shell	HCl	1 M	3 h	3 h	NaOH	5%	5, 15, 24 h	110	NaOH	1 M	3 days	70	4.3	—	153
Oyster shell	HCl	10%	3 days	3 days	NaOH	—	—	—	—	10%	24 h	30	69.65	—	154
*Acanthopleua vaillantii*	HCl	1 M	3 h	3 h	NaOH	5%	24 h	110	NaOH	1 M	—	70	—	—	153
Mussel shell	HCl	0.68 M	6 h	6 h	NaOH	25 M	20 h	75	NaOH	0.62 M	16 h	30	23.25	15.14	102
*Haliotis tuberculata*	25	—	HCl	HCl	30 min	NaOH	1 M	8 h	—	—	—	—	—	0.064	155
*Modiolus modiolus*	70	100	HCl	HCl	15 min	NaOH	1 M	20 min	110	NaOH	45%	24 h	40.13	10.21	150
*Bellamya javanica*	—	—	HCl	HCl	30 min	NaOH	4%	2 h	120	NaOH	60%	1 h	21.76	—	156
*Donax scortum*	—	80	HCl	HCl	24 h	NaOH	1 N	24 h	110	NaOH	40%	6 h	11.96	18.8	157
*Murex trapa*	25	70	HCl	HCl	2 days	NaOH	5%	2 days	90	NaOH	40%	6 h	—	17	158
*Anadara granosa*	—	—	HCl	HCl	2 days	NaOH	3.5 N	1 h	80	NaOH	50%	3 h	—	—	159
*Conus inscriptus*	60	80	HCl	HCl	30 min	NaOH	3 M	2 h	100	NaOH	50%	2 h	21.65	—	160
*Anadara granosa*	75	85	HCl	HCl	1 h	NaOH	3%	30 min	—	NaOH	45%	1 h	—	—	163
Snail shells	25	60	HCl	HCl	6 h	KOH	2%	24 h	105	NaOH	40%	2 h	—	—	161
*Perna viridis*	—	—	HCl	HCl	—	NaOH	1 N	—	25	NaOH	15%	24 h	41.6	39.5	162
*Monacha cantiana*	—	—	HCl	HCl	24 h	NaOH	2–4%	1 h	100	NaOH	50%	2 h	—	—	165
*Crassostrea iredalei*	75	80	HCl	HCl	2 h	NaOH	1 M	2 h	100	NaOH	50%	2 h	22.5	11.8	166
*Pomacea canaliculata*	25	65–100	HCl	HCl	2–3 h	NaOH	1–10%	2 days	100	NaOH	40–50%	—	—	17.48	167
*P. viridis*	25	70	HCl	HCl	2 days	NaOH	5%	2 days	90	NaOH	40%	6 h	—	18	168
*Telescopium telescopium*	25	70	HCl	HCl	2 days	NaOH	3%	2 days	—	NaOH	60%	4 h	42	—	169
*P. viridis*	25	70	HCl	HCl	2 days	NaOH	3%	2 days	—	NaOH	60%	3.5 days	—	—	170
*Doryteuthis singhalensis*	90	60	HCl	HCl	2 h	NaOH	—	3 h	90	NaOH	40%	2 h	37.65	85	171
*Amusium* sp*.*	90	90	HCl	HCl	2 h	NaOH	5%	140 min	90	NaOH	50%	—	—	9.7	172
*Doryteuthis sibogae*	—	80	HCl	HCl	24 h	NaOH	1 N	24 h	110	NaOH	40%	6 h	33.02	—	173
*S. kobiensis*	90	60	HCl	HCl	2 h	NaOH	—	3 h	90	NaOH	40%	2 h	29.87	43.77	174
*D. gigas*	—	25	—	—	—	NaOH	1 M	24 h	—	—	—	—	38.6	—	175
*S. officinalis*	24	90	HCl	HCl	—	NaOH	4%	20 min	140	NaOH	12.5 M	4 h	5	—	176
Squid pens	—	100	HCl	HCl	2 h	NaOH	10%	1 h	80	NaOH	50%	2 h	—	—	177
*Cuttlefish*	25	100	HCl	HCl	30 min	NaOH	1 N	1 h	90	NaOH	33%	10 min – 2 h	50	70	70

In contrast, 40% NaOH is used in the deacetylation process at 90 °C for two hours. 1 N HCl, 1 N NaOH, and 33% NaOH are the main chemicals used in cuttlefish sources, and the yield is 70%.

## 5. Cellulose

### 5.1 Chemical structure of cellulose

**Fig. 4:**
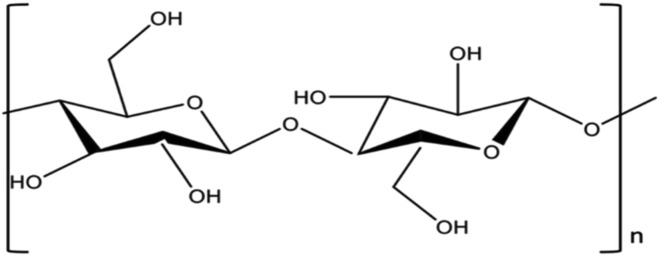
Chemical structure of cellulose.^180^

Cellulose is a carbon-rich material.^47^ It has axial carbon–hydrogen (C–H) planes that are hydrophobic and hydrophilic hydroxyl groups.^178^ It is present mainly in plant cell walls. This large-molecule polymer comprises repeated d-glucose units connected by β-1,4-glycosidic linkages (Fig. 4).^179^

There are crystalline and amorphous parts that make up cellulose.^47^ The primary chemical link that is widely distributed is the hydrogen bond, which forms a vast network and directly contributes to the crystalline structure.^179^ The amorphous part of cellulose can be hydrolyzed to separate the nanocrystals. Pure cellulose is hydrolyzed using strong acids at a regulated temperature and duration.^47^

### 5.2 Extraction of cellulose

#### 5.2.1 Extraction of cellulose from waste paper

Waste paper is another good source of cellulose. Old newspapers, recycled newsprint paper, office waste paper, *etc.*, can be used to synthesize cellulose.^54,181,182^ The deinking process on waste paper is done to remove ink and other undesirable contaminants except cellulose.^183^ Papers are shredded into pieces and converted into pulp before mixing the deinking agents in the pulp. This is done by washing or floatation and NaOH, H_2_O_2_, NaClO, NaClO_2_, surfactant sodium dodecyl benzene sulfonate (SDBS), and NaSiO_3_*etc.* are some common deinking agents (Table 6).^54,182,184,185^

**Table 6:** Common deinking agents and their parameters used in the extraction process of cellulose from waste paper

Sl no.	Deinking and purifying reagents	Strengths	Duration	Temperature	References
1	NaOH	5% (w/v)	Not defined	100 °C	54
	NaClO	2% (v/v)			
2	NaOH	2% (w/w)	30 min	53–57 °C	181
	Na_2_SiO_3_	2% (w/w)			
3	H_2_O_2_	1% (w/w)			
4	NaOH	0.5 and 1.0 M	5 h	Room temperature	184
	NaClO_2_	0.5, 1.0, and 2.0 M	1 h	75 °C	
5	NaOH	2.5% (w/w)	35 min	53–57 °C	183
	Na_2_SiO_3_	2.25% (w/w)			
	H_2_O_2_	0.50% (w/w)			
	NaClO_2_	1% (w/v)	1 h	75 °C	
	KOH	3.5% (w/v)	2 h	95 °C	
6	NaOH	1.5% (w/v)	30 min	Not defined	185
	H_2_O_2_	3% (w/v)			
	Na_2_SiO_3_	5% (w/v)			
	SDBS	1.5% (w/v)			
7	NaOH	1.2% (w/v)	1 h	45 °C	14
	Na_2_SiO_3_	1.8% (w/v)			
	C_18_H_34_O_2_	0.9% (w/v)			
	H_2_O_2_	0.8% (w/v)	50 min		
8	NaOH	5% (w/v)	2 h	125 °C	182
	NaClO	2% (w/v)	2 h	125 °C	
9	NaOH	2% (w/v)	2 h	90 °C	186
	NaClO_2_	Not defined	1 h	75 °C	
	KOH	2% (w/v)	2 h	90 °C	
10	NaOH	2% (w/v)	3 h	100 °C	187
	NaClO	2% (w/v)	1 h	70 °C	
11	CH_3_COOH	Not defined	1 h	70 °C	

The flotation technique is also used along with these deinking agents to purify and extract cellulose from waste papers. Air bubbles rise through the liquid in the tank with the suspended contaminant particles and waste paper pulp during the flotation deinking process. As the bubbles rise, ink clumps and hydrophobic impurities are gathered. After that, the adhered particles are shifted to a layer of foam, from which they can be readily removed.^181^ Tween-80 (0.1% w/w, 45 ± 2 °C for 7–12 min), commercial ISTEMUL 780 (0.1%), and sodium-4-polystyrene sulfonate (0.9%) are some floating agents used to form the froth layer.^14,181,183,188^ The airflow rate can be 10 L min^−1^ and a pressure of 3 bar.^14^ Besides the flotation tank, mechanical agitation can be applied by a hydrapulper or a repulper with a rate of 800–1000 rpm. This mechanical force, along with flotation and deinking agents, helps the ink fall off the waste paper and wash away other impurities like lignin and hemicellulose. Thus, pure cellulose is extracted from used papers.^183,185^

#### 5.2.2 Extraction of cellulose from waste fabric

Sample preparation: Supercritical CO_2_ (scCO_2_) is applied to clean and sterilize wasted cotton cloths, and a moderate temperature is maintained to remove microorganisms and impurities. Alkaline pulping : NaOH is used in a 1 : 20 ratio to remove lignin in this stage. This stage is important because it also helps it helps remove pectin and hemicellulose.^55^ Bleaching: hydrogen peroxide (H_2_O_2_) is used for decolorization of the pulped fiber. This step helps dissolve lignin and hemicellulose, enhancing the cellulose yield. Acid hydrolysis: 64 wt% H_2_SO_4_ is used in this step.^2^

Vanzetto *et al.* used cotton fabric (100% natural raw cotton) waste and polyester fabric (50% polyester fiber + 50% treated natural cotton fiber) as the source of cellulose. They cut the waste sample into pieces of 1 cm × 1 cm. The sample was treated by ultrafine friction milling for 6 hours after being submerged in water for 24 hours. They oxidized the milled textile residue with 2,2,6,6-tetramethylpiperidine-1-oxyl (TEMPO). TEMPO reagent was mixed with distilled water to prepare the initial solution. The textile sample was mixed with the initial solution, in which NaBr (99.9% w/w) and NaClO (12% v/v) were mixed. The pH of this solution was kept in the range of 10 to 10.5 with the help of 0.1 M NaOH solution. The mixture was stirred for 20 minutes after 5 mL of ethanol was added to it. The oxidized cellulose was separated by centrifuging.^13^

S. Thambiraj and D. Ravi Shankaran used industrial waste cotton to extract cellulose, convert the extracted cellulose fiber into cellulose microcrystals, and finally into cellulose nanocrystals. They cut the cotton sample into pieces and washed it with hot water. The washed cotton was then dried in an oven for 2 hours. The cotton sample was then treated by alkali hydrolysis. The cotton was heated under continuous stirring in 20% NaOH solution for 4 hours at 40–60 °C. Then water was added to the solution to neutralize the pH value. The neutral suspension was filtered, and the filtrate underwent hydrolysis to remove hemicellulose and lignin. 500 mL of 60% sulfuric acid was used in this acid hydrolysis. After the hydrolysis for 8 hours at 50–60 °C, the cellulose formed a white slurry. Again, water was added to this slurry to make it neutral. It was kept for 12 hours to settle down. After settling, the slurry was rewashed. Cellulose microcrystals were isolated from this slurry by centrifuging the suspension at 5000 rpm, forwarded by drying in an oven overnight at 60 °C, and purified with acetone.^189^

In Table 7, the sample was prepared using deionized water, supercritical CO_2_, and citric acid, and the temperature was kept between 60 and 100 °C. Different NaOH concentrations are employed in alkaline treatment. When combined with 3% CH_3_COOH and NaCl, NaClO_4,_ and H_2_O_2,_ it acts as a bleaching agent. In the acidic treatment, several amounts of H_2_SO_4_, HNO_3_, and HCl are often used.

**Table 7:** Preparation method conditions for cellulose fiber from textile cotton waste

Sl no.	Sample preparation			Alkaline treatment			Bleaching			Acidic treatment			References
	Chemicals	Time	Temp. (°C)	Chemicals	Time	Temp. (°C)	Chemicals	Time	Temp. (°C)	Chemicals	Time	Temp (°C)	
1	85 wt% citric acid	—	100	1 M NaOH	—	—	—	—	—	—	—	—	190
2	—	—	—	—	—	—	NaClO_4_ + NaCl	36 h	25	60 wt% H_2_SO_4_	1 h	25	191
							NaClO_2_ + H_2_O_2_ + NaCl	24 h	25				
3	—	—	—	3% NaOH	6 h	Boil	0.6% NaClO	30 min	Boil	68% (w/w) HNO_3_	1 h	60	192
										37% (w/w) HCl			
4	—	—	—	NaOH	4 h	25	—	—	—	64% H_2_SO_4_	1 h	45	193
5	Hot water	—	—	26% NaOH	90 min	170	2% NaClO_2_ + 3% CH_3_COOH	120 min	70	5% oxalic acid	6 h	—	55
							1.5% NaOH + 1% H_2_O_2_	90 min	70				
							1% NaClO_2_ + 3% CH_3_COOH	90 min	60				
6	Supercritical carbon dioxide	1 h	60	10 wt% (NaOH)	3 h	80	6 wt% (H_2_O_2_)	—	—	64 wt% H_2_SO_4_	1 h	45	2
7	Deionized water	10 min	25	10 wt% NaOH	2 h	70	1.5% (wt%) H_2_O_2_	—	—	98 wt% H_2_SO_4_ + 37 wt% HCl	7 h	55	194
8	Water	—	—	10% (w/w) NaOH	2 h	70	35% H_2_O_2_	3 h	45	35% w/w H_2_SO_4_	1 h	40	195

Chemical agents like NaClO, KOH, and Na_2_SiO_3_ pose environmental risks due to toxicity and alkaline waste, while H_2_O_2_ and oleic acid are greener alternatives. Proper reagent selection and wastewater management are essential for sustainable cellulose extraction.

#### 5.2.3 Extraction of cellulose from plant sources

**Fig. 5:**
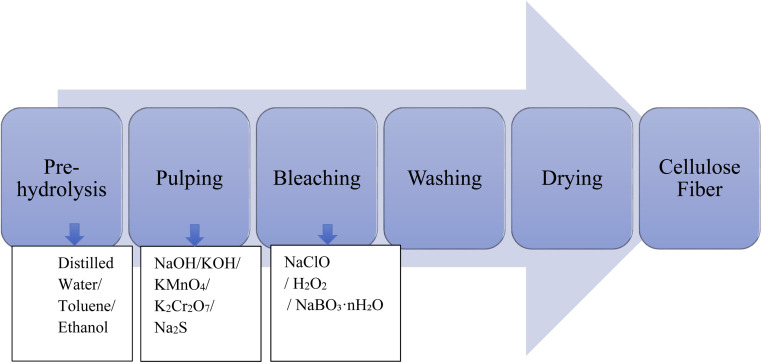
Flow sheet of the production of cellulose fiber from the plant source.

Pre-hydrolysis: first, the raw materials are washed with distilled water and cut into small pieces. Sometimes, toluene/ethanol (2 : 1, v/v) is used to remove wax, phenolics, pigments, and oils.^52^ Then NaOH or other alkali aqueous solution is used under a mechanical stirrer to remove other constituents present in the pulp.^49^ Pulping and Bleaching: there are two types of pulping treatment. Firstly, alkaline treatment, and secondly, acidic treatment. In alkaline treatment, plant materials are treated with an alkaline solution, typically sodium hydroxide, which helps to break down lignin from the cell wall.^196^ KOH, KMnO_4_, K_2_Cr_2_O_7_, Na_2_S, *etc.*, are also used in this step. The cellulose extraction process is described in Fig. 5. Then, the bleaching agent H_2_O_2_ is used to remove lignin and other non-cellulosic components.^197^ We can perform an additional bleaching step using another agent like sodium hypochlorite (NaClO) or sodium perborate (NaBO_3_·*n*H_2_O) to achieve a higher degree of purity and whiteness. An acidic treatment sometimes follows bleaching steps to neutralize the alkaline residues and further purify the cellulose. Typically, H_2_SO_4_ is used in this step.^198^

Anuj Kumar and Yuvraj Singh Negi used sugarcane to extract cellulose nanocrystallites.^199^ They first extracted the cellulose nanofibers by following the method done by others. This process requires less time for production than the conventional processes. At first, lignin was removed from the sample with the help of sodium chlorite solution. After treatment with sodium chlorite, the resultant white substance was treated with 3 wt% and 6 wt% of potassium hydroxides at 80 °C for 2 hours to leach hemicellulose, pectin, and starch. After this, the purified cellulose fiber was soaked in distilled water and placed in an ultrasonic generator at 20–25 kHz. After 30 minutes of ultrasonication, the nanofibers of cellulose were isolated. Cellulose nanocrystals were then extracted from cellulose nanofibers by acid hydrolysis. This hydrolysis was done with the help of sulfuric acid (64% w/w) solution at 45 °C for 60 minutes to isolate the cellulose nanocrystals from cellulose microcrystals, they used a method similar to Anuj Kumar and Yuvraj Singh Negi.^199^ Except, they heated the microcrystals with the sulfuric acid solution for 30–180 minutes, and the solution was centrifuged for 30 minutes.^189^ Bibin Mathew Cheriana *et al.* used pineapple leaves as the source of cellulose.^200^

To scale up cellulose production in the industrial sector and increase production efficiency, Sakinul Islam *et al.* used the high-pressure process (HPP) and low-pressure process (LPP). They used rice husk from the paddy mill as the source of cellulose. The delignification process was done under LPP and HPP. They concluded that removing amorphous components like lignin, hemicellulose, *etc.*, was more efficient in HPP. The cellulose content was higher (93.68%) in HPP than in LPP (75.23%).^201^

Hemmati, F. *et al.* used walnut shells as the cellulose fiber source. They slightly modified the method proposed by Bano and Negi to extract cellulose from the walnut shell. The walnut shell was ground and sieved before undergoing the dewaxing process. They used a methanol and benzene solution (ratio of 20 : 80) for 6 hours. They used a 1 M sodium hydroxide solution under continuous stirring for 4 hours at 85 °C to remove hemicellulose. After rinsing with distilled water several times, they used acetic acid and sodium chlorite to maintain the pH level at 3–4.^202^

Xiao-Feng Sun and Run-Cang Sun used a chlorine-free method to extract cellulose from straw. This method is more environmentally friendly than other conventional methods. They dewaxed dried straw in a toluene-ethanol solution. The dewaxed straw was then preheated with NaOH in 60% methanol (0.5 M, 60 °C, 2.5 hours). Then it was post-treated with 2% H_2_O_2_ and 0.2% TAED (tetra acetylene diamine) (48 °C, pH 11.8, 12 hours). The crude cellulose was purified with 80% acetic a and 70% nitric acid.^203^ Their method creates less pollution than the conventional methods, but it is more time-consuming and costly. The size distribution of cellulose fiber was lower in the conventional method than in the chlorine-free method.^27^

In Table 8, carrot peel (81%) and pineapple leaves (81.27%) have the highest cellulose content. Pretreatment of pineapple leaves involves a 2% detergent solution at 70 °C, followed by bleaching with 10% NaClO at 100 °C and 17.5% NaOH. 2% NaOH is applied for three hours at 80 °C during the pulping stage. Carrot peel cellulose is extracted using an aqueous acetic buffer containing 1.7% aq. The bleaching step uses a chlorite solution, while the pulping step uses 2% NaOH at 80 °C for three hours. Jackfruit peel contains the least quantity of cellulose (20.08%). This source is pretreated with 1 M NaOH and bleached with 1.5% NaClO_2_ at 70 °C for two hours. In the pulping stage, 65% H_2_SO_4_ is used for 1 h at 37 °C.

**Table 8:** Extraction conditions of cellulose from different plant sources

Sl no.	Sources	Cellulose (%)	Pre-hydrolysis			Bleaching			Pulping			Ref.
			Chemicals	Temp. (°C)	Time	Chemicals	Temp. (°C)	Time	Chemicals	Temp. (°C)	Time	
1	Rice husk	35	5 wt% NaOH	Boil	24 h	1% (w/v) NaClO_2_	Boil	2 h	5% (w/v) Na_2_SO_4_	—	1 h	1
									18% (w/v) KOH	25	48 h	
									75% (wt) H_2_SO_4_	—	10 min	
2	Rice husk	33	4 wt% NaOH	Reflux	2 h	Buffer solution of CH_3_COOH, aq. Chlorite	100–130	4 h	10 M H_2_SO_4_ centrifugation at 10 000 rpm	10	10	204
3	Rice husk	36–40	5 wt% NaOH	120	45 min	NaClO_2_ + CH_3_COOH	70	5 h	5% H_2_SO_4_ + 5% H_2_O_2_ + 25% CH_3_COOH	120	2 h	205
									10% HNO_3_ + 5% H_2_O_2_ + 25% CH_3_COOH			
4	Rice husk	∼35	Deionized water	50	24 h	—	—	—	4% (w/w) H_2_SO_4_	Reflux	2 h	88
									5% (w/w) NaOH	Reflux	2 h	
									3% (w/w) NaCl	75	4 h	
5	Rice straw	30–60	2 wt% NaOH	170	3 h	NaClO_2_, CH_3_COOH	—	—	18% NaOH	170	3 h	24
6	Sugarcane bagasse	40–50	C_2_H_5_OH and deionized water mixture	100	1.5 h	1% NaOCl	95	60 min	0.5, 1.5, 2.75 and 4% NaOH	120	15, 30, 45 min	25
7	Sugarcane bagasse	40–50	C_2_H_5_OH and deionized water mixture	100	1.5 h	NaOH–H_2_O_2_	105	24 h	Dilute H_2_SO_4_ (0.5, 2.5, 5%)	—	15–30 min	25
8	Cotton stalk	—	0.1% H_2_SO_4_	160	30 min	10% NaClO, 2.2% H_2_O_2_	60	60	14–20% NaOH + Na_2_S	170	90 min	26
9	Sisal fiber	50–74	0.1 M NaOH in 50% volume C_2_H_5_OH	45	3 h	0.5%,1%,2%,3% H_2_O_2_	45	3 h	10% NaOH − 1% Na_2_B_4_O_7_·10H_2_O	28	15 h	27
									70% HNO_3_ + 80% HAc	120	15 min	
10	Sisal fiber	50–74	0.1 M NaOH	45	3 h	0.7% NaClO_2_	Boil	2 h	17.5% NaOH			27
11	Leaves (*Pamdanus tectorius*)	37.3 ± 0.6	Stagnant water	100	15	1.7% NaClO_2_	125	4 h	4% NaOH	125	4 h	28
12	Kenaf	—	—	—	—	Acetate buffer (NaOH in CH_3_COOH) + NaClO_2_	90	4 h	4% NaOH	90	3 h	29
13	Kenaf stalk	56.81	NaOH	—	—	Alkaline–H_2_O_2_ 50% purify	85	2 h	95–98% purified, 4% H_2_SO_4_	140	1 h	29
									0.02% KMnO_4_	25	20 min	
						Alkaline–NaClO_2_ 77.5–82.5%	70	2				
									0.02% K_2_Cr_2_O_7_	25	2 h	
14	Ramie plant	—	Deionized water	Sundry	3 days	5% NaClO_2_ + CH_3_COOH	170	120 min	18% NaOH	170	120 min	206
						4% KOH	80	60 min				
15	Jute	60.79	2 wt% NaOH	60	2 days	30% H_2_O_2_	25	Overnight	27.7% H_2_SO_4_	45	30 min	30
16	Unripe coconut husk	32.5	2 wt% NaOH	80	2 h	NaClO_2_ in Glacial acetic acid	60–70	—	0.05 N HNO_3_	70	1 h	207
17	Coconut coil	—	60% w/w C_2_H_5_OH	150	4 h	3% H_2_O_2_ + 4% NaOH	50	180 min	70% C_2_H_5_OH	—	—	31
18	Pineapple crown leaves	79–83	1 M NaOH	80	1 h	H_2_O_2_	80	1 h	3 M H_2_SO_4_	45	1, 2, 3 h	32
19	Pineapple leaves	71.5	Deionized water	80	—	10% NaClO	—	—	5% NaOH	—	—	33
20	Pineapple leaves	81.27	2% detergent solution	70	—	10% NaClO	100	30	2% NaOH	80	3 h	208
						17.5% NaOH	80	1 h				
21	Banana peel	—	1% potassium metabisulfite	60	24	1% NaClO_2_ + 10% CH_3_COOH	70	1 h	20% NaOH + 0.1% anthraquinone	170	1.5 h	34
22	Alfa grass	—	3 N NaOH	100	2 h	50% NaClO	80	48 h	50% v/v H_2_SO_4_	70	30 min	35
23	Municipal grass	—	Distilled water	100	60 min	5% (v/v) H_2_O_2_ + 1.3% NaOH + 0.7% NaClO_2_	80	90 min	4% NaOH	Reflux	90 min	36
24	African napier grass	47.1	Toluene–ethanol (2 : 1 v/v)	110	6 h	NaClO_2_ + CH_3_COOH + 2% Na_2_S	100	2 h	17.5% NaOH	20	45	37
									10% CH_3_COOH			
25	Native yellow thatching grass	29.64 ± 0.81	Toluene–ethanol (2 : 1 v/v)	110	4 h	NaClO_2_ + CH_3_COOH	95	6 h	10% NaOH	100	4 h	209
26	Sabai grass	>55	Water	Sundry	3–4 days	H_2_O_2_ + 99.8% glacial acetic acid	130	3 h	6% NaOH	80	2 h	38
27	Bamboo	—	Toluene–ethanol (2 : 1 v/v)	250	2 h (10–12) cycles	35% H_2_O_2_ + 99.8% CH_3_COOH in presence of TiO_2_	130	2 h	6% NaOH	80	2 h	39
28	Barks of mulberry (*Morus alba* L.)	37.38 ± 2.31	1 (w/v) % NaOH	80	2 h	0.7 (v/v) % NaClO_2_ with acetate buffer + NaOH–glacial acetate acid	80	1.5 h	1 w/v% NaOH + 1 w/v% Na_2_S and a bath ratio of 1 : 30	80 and 130 respectively	1.5 h	40
29	Peel of prickly pear fruits	27	38 : 62 (v/v) Toluene–ethanol	Reflux	24 h	—	—	—	0.5% ammonium oxalate	60	2 h	210
									0.05 N HCl	80	21 h	
									2% NaOH	80	2 h	
30	Wheat straw	45.70 ± 0.18	2% solution of NaOH		Over night	8 (v/v) % H_2_O_2_	25	Overnight	10% HCl (1 N) solution	60 ± 1	5 h	41
			10–12 wt% NaOH	200 ± 5	4 h							
31	*Luffa cylindrica*	—	4% (w/w) NaOH	80	2 h	—	—	—	45% H_2_SO_4_	50	40 min	48
32	Corncob	31.2 ± 3.1	2% (w/w) NaOH	100	4 h	Acetate buffer (NaOH + glacial acetic acid), 1.7 wt% NaClO_2_	80	6 h	9.17 M H_2_SO_4_	45	30, 60, 90 min	42
33	Poplar trees wood	—	2 : 1 (v/v) mixture of benzene/ethanol	90	6 h	NaClO_2_	75	1 h	3 wt% KOH	80	2 h	43
									6 wt% KOH	80	2 h	
34	Soybean	—	17.5% NaOH	25	2 h	Chlorine dioxide solution	50	1 h	1 M HCl	80	2 h	44
									2% (w/w) NaOH			
35	Lemon peel	—	—	—	—	NaOH and H_2_O_2_	High temp	—	2.5 N HCl	Reflux	10 h	45
36	Henequen fiber	60	0.4% H_2_SO_4_	Boil	—	3.5% NaClO	30	—	20% NaOH	25	—	211
37	Jackfruit (*Artocarpus heterophyllus*)	—	2% NaOH	50	180 min	3% H_2_O_2_ + 4% NaOH	50	180 min	52% (w/w) H_2_SO_4_	45	120 min	209
38	Jackfruit peel	20.08 ± 0.05	1 M NaOH	60	2 h	1.5% NaClO_2_	70	2 h	65% H_2_SO_4_	37	1 h	46
39	Palm oil empty fruit bunches	36.67	Soaked in distilled water	25	24 h	—	—	—	55%, 60%, and 64% H_2_SO_4_	35 and 45	3 h and 6 h	47
40	Onion skin	81	Distilled water	85	2 h	Acetate buffer + 1.7% aq. Chlorite	80	2h	2% NaOH	80	2 h	212
41	Carrot peel	41.1 ± 1.1	Water + air oven	105	24 h	0.7% NaClO_2_ + 5% CH_3_COOH + 5% Na_2_S	Boil	5h	17.5% NaOH	20	45	49
42	Pumpkin peel	—	2 wt% NaOH	100	4 h	0.5 M NaOH + 2% (v/v) H_2_O_2_	55	5 h	1-Butyl-3-methylimidazolium chloride	90	24 h	50
43	Tomato peel	—	Toluene/ethanol (2 : 1, v/v)	70	24 h	1.4% NaClO_2_	70	5 h	5% KOH	90	2 h	51
						NaOH/4% H_2_O_2_	90	5 h	NaOH	45	6 h	
44	Potato residues	24.86	Deionized water	Boil	3 min	10% (v/v) H_2_O_2_	70	1.5 h	7% NaOH	70	1 h	52
45	Abaca pulp	66.43	2 wt% of sodium chlorites + KCl, HCl buffer solution	—	Over night	NaClO_2_	—	—	Na_2_CO_3_–NaHCO_3_ buffer solution + NaBr (1.0%) + TEMPO (0.16%)	—	—	53
46	*Juncus effusus*	40	Distilled water	60	Over night	H_2_O_2_, NaOCl, NaBO_3_·4H_2_O	95	45 min	8 M NaOH	100	3 h	197
47	Pine trees	—	Deionized water	—	1 h	30% (w/w) H_2_O_2_	50	1 h	2% (w/v) NaOH	120	1 h	55
						1 M H_2_SO_4_ + 1 M, 10% (v/v)	95	1 h				
						1-Ethyl-3-methylimidazolium chloride						
48	Orange peel	—	Ethanol and toluene	—	—	0.7% (w/v) NaClO_2_	80	2 h	NaOH/KOH	Reflux	2 h	179

## 6. Conclusion

Utilizing natural and waste-derived resources, such as crab shells, plant leftovers, textile scraps, and newspaper waste, to extract cellulose, chitin, and chitosan has become more important due to the increasing demand for eco-friendly and sustainable products. The basic chemical and biological extraction methods of chitin and chitosan have been described in this study, focusing on the steps of demineralization, deproteinization, and deacetylation. Because of their increased production and efficiency, chemical methods continue to govern industrial applications and pose environmental challenges. On the other hand, although being ecologically safe, biological approaches are limited by their higher prices, longer processing times, and lower efficiency. A potential approach for recovering plentiful textile and agricultural waste is cellulose extraction, which involves a series of steps such as pre-hydrolysis, pulping, bleaching, and washing. Reagent use and energy input, however, continue to be significant issues that necessitate more environmentally friendly options. Future studies should focus on developing microbial strains and enzyme engineering to enhance the efficiency and reduce the cost of biological extraction methods. Incorporating sustainable reagents and improving hybrid approaches that combine the advantages of chemical and biological processes must also be priorities. These developments will widen the possibility for greater industrial use and significantly support a circular economy by bridging the gap between the generation of waste and resource recovery.

## Conflicts of interest

There are no conflicts to declare.

## Data Availability

Data will be made available on request from authors.
